# Positive association between serum bilirubin within the physiological range and serum testosterone levels

**DOI:** 10.1186/s12902-024-01651-z

**Published:** 2024-07-18

**Authors:** Cunbao Ling, Yadong Liu, Meiling Yao, Libing Tian

**Affiliations:** 1 School of Basic Medical Sciences, Jiangsu Vocational College of Medicine, Yancheng, Jiangsu Province China; 2 Department of Urology, The Yancheng School of Clinical Medicine of Nanjing Medical University, Yancheng, Jiangsu Province China; 3 Department of Urology, The sixth affiliated hospital of Nantong University, Yancheng, Jiangsu Province China; 4 School of Public Health and Management, Jiangsu Vocational College of Medicine, Yancheng, Jiangsu Province China

**Keywords:** Testosterone deficiency, Serum bilirubin, A population-based study

## Abstract

**Backgrounds:**

Research has demonstrated that elevated serum total bilirubin (STB) levels have a beneficial impact on various diseases, particularly metabolic syndrome. This study aims to investigate the association between STB levels and serum testosterone (STT) in order to determine if bilirubin plays a protective role in relation to testosterone deficiency (TD) risk.

**Methods:**

In this study, a total of 6,526 eligible male participants aged 20 years or older were analyzed, all of whom took part in the National Health and Nutrition Examination Survey (NHANES) conducted between 2011 and 2016. To investigate the relationship between STB and STT levels, we employed weighted multivariate regression models along with restricted cubic splines (RCS). Additionally, a subgroup analysis was conducted to explore the heterogeneity of this relationship across different subpopulations.

**Results:**

Among the participants, 1,832 individuals (28.07%) were identified as having testosterone deficiency (TD), defined as an STT level below 300 ng/dL. A significant positive correlation between STB and STT levels was observed in both crude and adjusted models (all *P* < 0.0001). The association between STB and STT levels was found to be statistically significant up to a threshold of 17.1 µmol/L, after which it became statistically insignificant(*P* for non-linearity = 0.0035). Weighted logistic regression analysis indicated that a 1 µmol/L increase in STB was associated with a 4% decrease in the likelihood of TD (odds ratio = 0.96, *P* < 0.0001). Subgroup analysis showed that the inverse relationship was limited to individuals aged 60 and over, non-smokers/drinkers, and obese individuals.

**Conclusion:**

STB within the physiological range(17.1 µmol/L) was positively associated with STT in adult males. The potential protective role of bilirubin regarding testosterone levels merits further exploration.

## Introduction

Testosterone (TT) in males is primarily produced by the Leydig cells, with contribution from the adrenal glands. During pre-adulthood, TT is crucial for the growth and development of males. As individuals age, there is a natural decline in TT production, which may lead to the development of testosterone deficiency (TD) or hypogonadism. Starting at age 30, TT levels decrease approximately by 1% each year [1]. Around 30% of men between the ages of 40 and 79 are affected by TD [2]. Besides aging, factors like obesity, dyslipidemia, metabolic syndrome, hypertension, diabetes mellitus, and chronic kidney disease have been linked to decreased TT levels and an elevated risk of developing TD [3–6]. TD can result in muscle atrophy, reduced strength, compromised cognitive function, poorer sleep quality, an increased risk of depression, and reduced life satisfaction in men [7, 8]. Understanding the factors leading to this decline is essential for preventing TD and developing new treatments.

Bilirubin, a tetrapyrrolic compound resulting from heme catabolism, has been shown in multiple studies to provide health benefits within a physiological range through antioxidation, anti-inflammation, promotion of metabolic health, and regulation of the immune system [9, 10]. Recent research suggests that bilirubin acts similarly to a hormone, affecting various cellular signaling pathways [11, 12]. Both animal-derived bilirubin, such as that from cow bezoar, and plant-derived bilirubin analogs, like phycocyanin, have been shown to exert beneficial effects on human health [13–15]. Moreover, taking iron and zinc supplements or adjusting dietary strategies can gently increase bilirubin concentration [16, 17].

To date, limited research has been conducted on the correlation between bilirubin and testosterone levels, with only one study addressing this topic [18]. This study, involving 1,284 men from a single medical center, found an independent and inverse association between serum bilirubin level and TD, exhibiting a dose-response relationship [18]. In this study, serum bilirubin level was categorized into quartiles. The overall prevalence of TD significantly decreased as the serum bilirubin quartile increased. Low bilirubin levels may be a novel risk factor for TD. Our study aims to explore this association in adult males by utilizing data from a general population-based survey. Furthermore, we examined the potential non-linear correlation between STB and STT by using the restricted cubic splines (RCS) model.

## Methods

### Data source

The data analyzed were obtained from the NHANES survey cycle spanning from 2011 to 2016(https://wwwn.cdc.gov/Nchs/Nhanes/). NHANES stands for the National Health and Nutrition Examination Survey, a biennial nationwide survey aimed at gathering comprehensive health and dietary information from a representative sample of the non-institutionalized U.S. population. The survey examines approximately 5,000 individuals annually, drawn from diverse counties across the United States. These individuals are grouped into a total of 30 primary sampling units (PSUs), with half of them being visited each year [19]. Distinctive in its approach, NHANES integrates interviews, physical assessments, and laboratory tests to generate extensive quantitative and qualitative data. Before any data collection commenced, all participants were required to provide written informed consent following the stipulations of the Public Health Service Act.

### Study population

Since 1999, NHANES has been publishing its data every two years. However, comprehensive data on TT measurements were only released during three specific cycles between 2011 and 2016. Thus, we have included 29,902 participants from three continuous cycles (2011–2012, 2013–2014, and 2015–2016).

In this research, we included the independent variable of serum TT levels and the dependent variable of serum bilirubin levels. For our subsequent analysis, we incorporated various covariates, including age, race, marital status, education level, alcohol consumption, smoking habits, body mass index, diabetes, and hypertension.

**Fig. 1:**
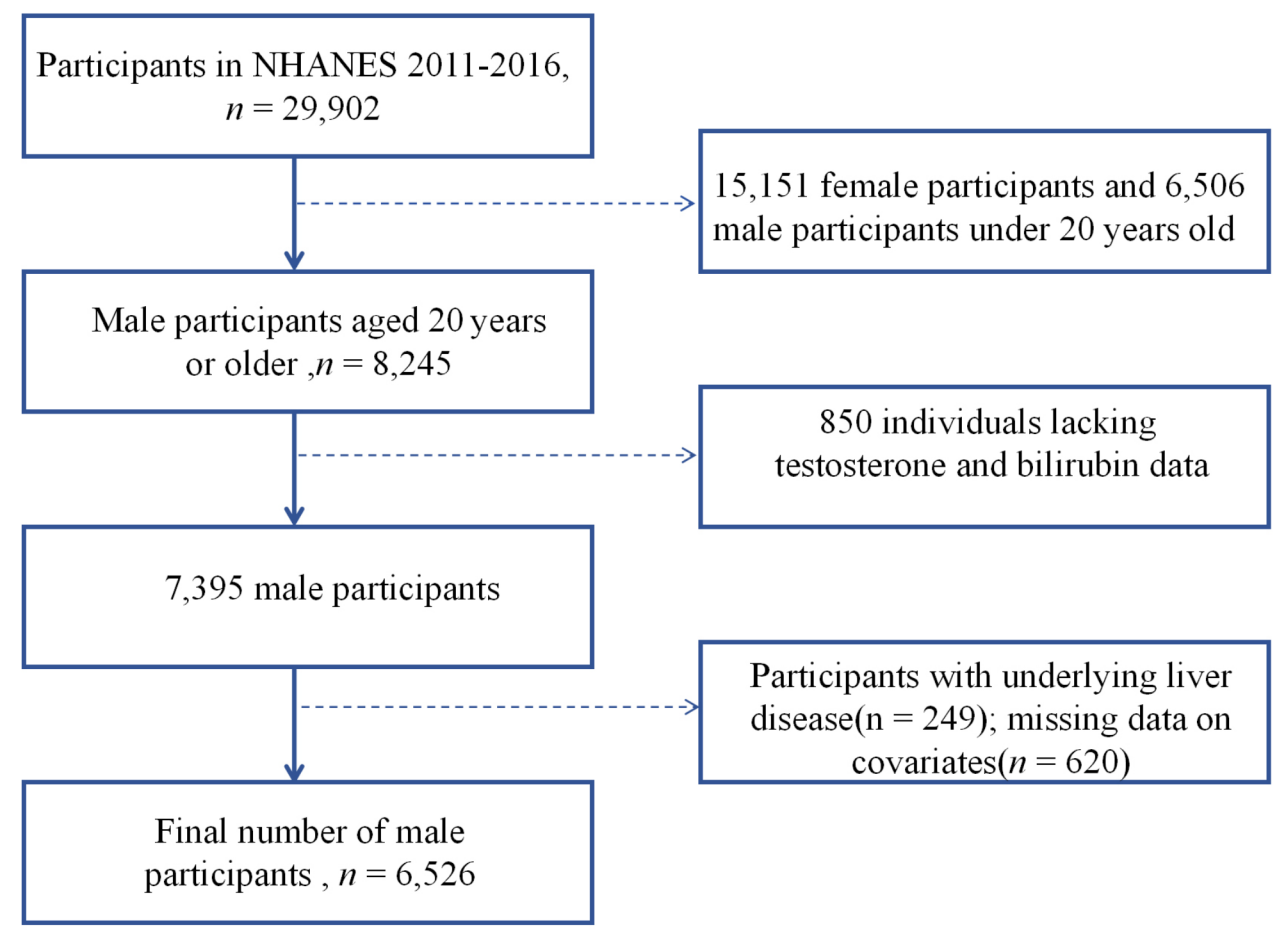
The flowchart of participants’ selection

We established the following exclusion criteria for selecting eligible participants: (1) male participants younger than 20 years old due to their relatively unstable testosterone levels (*n* = 6,506) and female participants (*n* = 15,151); (2) participants with missing or insufficient information on testosterone and bilirubin levels (*n* = 850); (3) liver disease not only affects hormone metabolism but also serves as a major factor influencing bilirubin levels, so we excluded individuals with potential liver disease(*n* = 249). Potential liver disease was defined as having total bilirubin levels exceeding 34.2 µmol/L, aspartate aminotransferase levels above 80 IU/L, or a self-reported history of liver disease; and (4) participants with missing data on covariates (*n* = 620). After applying these criteria, we narrowed down our research population to 6,526 male participants out of the initial 29,902 for further analysis (Fig. 1).

### Detection of bilirubin and TT levels

To ensure consistency and minimize biological fluctuations, blood samples were collected in the morning following an overnight fast. These specimens were then transported frozen on dry ice for immediate analysis or stored at − 80 °C for optimal preservation upon arrival.

To measure total serum testosterone levels, the Centers for Disease Control and Prevention (CDC) developed isotope dilution-liquid chromatography-tandem mass spectrometry (ID-LC-MS/MS) for routine analysis, with the lowest linearity limit of 0.75 ng/dL [20]. This methodology was designed for processing a high number of samples efficiently and has demonstrated exceptional accuracy and precision over a prolonged period. It is officially certified by the CDC Hormone Standardization Program (HoSt). Additional information regarding the laboratory methodology is available online (https://wwwn.cdc.gov/Nchs/Nhanes/2015-2016/TST_I.htm#Laboratory_Method_Files). The American Urological Association (AUA) advises clinicians to use a total testosterone level of less than 300 ng/dL as a suitable threshold for supporting the diagnosis of TD( Evidence Level: Grade B) [21].

Bilirubin concentration was assessed using an automated biochemical analyzer (DXC800, Beckman, USA), which applies the timed-endpoints method (Jendrassik-Grof) to determine the total bilirubin concentration in serum or plasma. In this assay, bilirubin interacts with a diazo reagent in the presence of caffeine, benzoate, and acetate, leading to the formation of azobilirubin. The change in absorbance at 520 nm over a specific time interval is directly proportional to the total bilirubin concentration.

### Definition of covariates

To assess the potential influence of various factors on the outcomes, we identified several variables that may serve as confounding factors. These variables include age (quantified in years), race (categorized as non-Hispanic white, non-Hispanic black, Mexican American, or other), marital status (categorized as married/living with partner, widowed/divorced/separated, or never married), education level (categorized into less than high school, high school, or beyond high school), and alcohol use (categorized as never, former, or current). Smoking status was categorized into three groups: never (individuals who have smoked fewer than 100 cigarettes in their lifetime), former (individuals who have smoked more than 100 cigarettes in their lifetime but currently do not smoke), and current (individuals who have smoked more than 100 cigarettes in their lifetime and currently smoke on some days or every day). Body Mass Index (BMI) was calculated as weight divided by the square of height and categorized into normal (BMI < 25 kg/m^2^), overweight (25 ≤ BMI ≤ 29.9 kg/m^2^), or obesity (≥ 30 kg/m^2^). Hypertension was determined by a physician’s diagnosis, a systolic blood pressure ≥ 140 mm Hg, diastolic blood pressure ≥ 90 mm Hg, or use of antihypertensive medication. Diabetes was identified by a physician’s diagnosis, fasting plasma glucose ≥ 7.0 mmol/L, HbA1c ≥ 6.5%, random blood glucose ≥ 11.1 mmol/L, or anti-hyperglycemic medication use. This comprehensive categorization elucidates the potential confounders affecting the study’s results.

### Statistical analyses

We conducted data analysis in accordance with the NHANES analytic guidelines. We utilized the Mobile Examination Center (MEC) examination weight (WTMEC2YR) for weighted analysis because some variables were collected at the MEC. The sample weight for the final analysis was adjusted to WTMEC2YR/3, representing the amalgamation of three NHANES survey cycles.

Continuous variables were reported as mean and standard error (SE), while categorical variables were presented as numbers and percentages. We compared baseline characteristics between subjects with and without TD using the t-test for continuous variables and the χ^2^ test for categorical variables. We then examined the association between STB and STT levels using multivariable weighted linear regression models and assessed the relationship between STB and TD via multivariable weighted logistic regression models. Tertiles of STB were established and considered as categorical variables in the regression analyses. We presented non-adjusted, partially adjusted (accounting for age, marital status, and race), and fully adjusted models (including adjustments for age, marital status, race, educational level, smoking status, alcohol use, BMI, diabetes, and hypertension). To detect potential nonlinear relationships between STB and STT levels, Restricted Cubic Spline (RCS) functions were employed, and the analysis was conducted using the rms package in R. By introducing flexibility through the use of spline functions, RCS can capture nonlinear and non-monotonic relationships that may be overlooked by traditional parametric methods. Furthermore, a subgroup analysis was performed utilizing R package ‘forestplot’, aiming to detect the heterogeneity of this relationship among diverse subpopulations. All statistical procedures were executed using R version 4.2.0. A two-sided p-value < 0.05 was deemed to indicate statistical significance.

## Results

### Baseline characteristics of research participants

The American Urological Association (AUA) has established a serum level below 300 ng/dL as the threshold for defining testosterone deficiency (TD). Table 1 presents the weighted distribution of baseline characteristics for a selected U.S. cohort, divided into two groups based on the presence of TD. The cohort consists of 4,694 participants without TD and 1,832 (28.07%) individuals affected by it. Individuals with TD exhibited significantly lower mean STB levels, averaging at 11.31 ± 0.14 µmol/L, in contrast to those without TD, who demonstrated levels of 12.29 ± 0.17 µmol/L. This difference was statistically significant, with a p-value of less than 0.0001. The prevalence of TD in individuals aged 60 and above is significantly higher compared to those under 60 (33.63% vs. 25.53%, p-value = 0.003). Among obese individuals, the prevalence of TD is 43.27%, which is higher than in overweight and normal BMI individuals (25.48% and 13.07% respectively, p-value < 0.0001). Subgroups living with a partner, former smokers, and former drinkers also showed significantly higher TD prevalence rates (all p-values < 0.0001). Additionally, individuals with diabetes and hypertension had elevated TD prevalence rates (42.38% and 34.41% respectively).

**Table 1:** Baseline characteristics of the participants

Variables	Total	Non-testosterone deficiency	Testosterone deficiency	*P*
	*N* = 6,526	*N* = 4,694(71.93)	*N* = 1,832(28.07)	
Age (years), mean (SE)	47.24 (0.37)	46.14 (0.44)	50.21(0.54)	< 0.0001
BMI (kg/m^2^), mean (SE)	28.89 (0.12)	27.70 (0.11)	32.10(0.27)	< 0.0001
STT (ng/dL), mean (SE)	415.24 (3.37)	485.59(3.06)	224.91(1.66)	< 0.0001
STB (µmol/L), mean (SE)	12.03 (0.15)	12.29(0.17)	11.31(0.14)	< 0.0001
Age (years)				0.003
<60	4477(75.80)	3334(74.47)	1143(25.53)	
≥60	2049(24.20)	1360(66.37)	689(33.63)	
Race, n (%)				0.21
Mexican American	877 (8.70)	633 (72.18)	244(27.82)	
Non-Hispanic Black	1394 (9.72)	1031 (73.96)	363(26.04)	
Non-Hispanic White	2621 (67.80)	1846 (70.43)	775(29.57)	
Other	1634 (13.79)	1184 (72.46)	450(27.54)	
Marital status, n (%)				< 0.0001
Married/living with partner	4205 (66.97)	2906 (69.1)	1299(30.9)	
Widowed/divorced/separated	997 (12.45)	713(71.51)	284(28.49)	
Never married	1324 (20.57)	1075 (81.19)	249(18.81)	
Education level, n (%)				0.78
≤High school level	3013 (37.59)	2151 (71.39)	862(28.61)	
> High school level	3513 (62.41)	2543 (72.39)	970(27.61)	
Smoke status, n (%)				< 0.0001
Never	3073 (49.35)	2216 (72.11)	857(27.89)	
Former	1941 (29.53)	1282 (66.05)	659(33.95)	
Now	1512 (21.12)	1196 (79.10)	316(20.90)	
Alcohol use, n (%)				< 0.0001
Never	598 (7.29)	408 (68.23)	190(31.77)	
Former	1173 (14.65)	777 (66.24)	396(33.76)	
Now	4755 (78.06)	3509 (73.8)	1246(26.20)	
BMI, n (%)				< 0.0001
Normal	1829 (25.97)	1590 (86.93)	239(13.07)	
Overweight	2469 (38.16)	1840 (74.52)	629(25.48)	
Obesity	2228 (35.87)	1264 (56.73)	964(43.27)	
Diabetes, n (%)				< 0.0001
No	5200 (84.39)	3930 (75.58)	1270(24.42)	
Yes	1326 (15.61)	764 (57.62)	562(42.38)	
Hypertension, n (%)				< 0.0001
No	3713 (60.77)	2846 (76.65)	867(23.35)	
Yes	2813 (39.23)	1848 (65.69)	965(34.31)	

*Footnotes* Continuous variables were reported as mean and standard error (SE), while categorical variables were presented as numbers and percentages

*Abbreviations* BMI, body mass index; STT, serum testosterone; STB, serum total bilirubin

### Relationship between STB and STT

Following a weighted multivariate linear regression analysis (Table 2), a significant positive correlation between STB and STT levels was identified. This association remained consistent across the unadjusted model (β = 3.69; 95% CI = 2.62–4.76, *P* < 0.0001), the partially adjusted model (β = 3.52; 95% CI = 2.50–4.55, *P* < 0.0001), and particularly in the fully adjusted model (β = 2.5; 95% CI = 1.47–3.53, *P* < 0.0001), indicating a 2.5 ng/dL increase in STT for each µmol/L increase in STB. Additionally, the highest tertile (T3) exhibited the highest serum TT levels compared to the lowest tertile (T1). β-values indicated significant increases across models: 37.1 (95% CI = 24.72–49.48, *P* < 0.0001) in the unadjusted model, 37.22 (95% CI = 25.22–49.21, *P* < 0.0001) in the partially adjusted model, and 27.93 (95% CI = 16.12–39.74, *P* < 0.0001) in the fully adjusted model. The significant p-value for trend (< 0.0001) across all models highlights a dose-response relationship between STB and TT levels, confirming the strength and consistency of this association. Weighted logistic regression analysis indicated that across all models (Table 3), a one µmol/L increase in STB was associated with a 4% decrease in the likelihood of TD [odds ratio (OR) = 0.96, *P* < 0.0001]. Segmented regression models exhibited a consistent pattern across intervals of STB (p-value for trend < 0.0001).

**Table 2:** Association between STT and STB in multiple survey-weighted generalized linear regression models

Bilirubin level	Unadjusted model		Partially adjusted model		Fully adjusted model	
	β(95%CI)	*P*	β(95%CI)	*P*	β(95%CI)	*P*
Continuous variable	3.69 (2.62,4.76)	< 0.0001	3.52 (2.50, 4.55)	< 0.0001	2.5 (1.47, 3.53)	< 0.0001
Categorical variable						
Tertile 1	1 (Ref)		1 (Ref)		1 (Ref)	
Tertile 2	28.37 (15.57,41.16)	< 0.0001	29.45 (16.24,42.66)	< 0.0001	22.84 (10.93, 34.76)	< 0.001
Tertile 3	37.1 (24.72,49.48)	< 0.0001	37.22 (25.22,49.21)	< 0.0001	27.93 (16.12, 39.74)	< 0.0001
P for trend		< 0.0001		< 0.0001		< 0.001

*Footnotes* Tertile 1: < 10.26µmol/L; Tertile 2: 10.26–13.68µmol/L; Tertile 3: 13.68–34.2µmol/L. The partially adjusted model was controlled for age, marital status, and race. The fully adjusted model was controlled for age, race, marital status, education, body mass index, smoking, alcohol consumption, diabetes, and hypertension

**Table 3:** Association between TD and STB in multiple survey-weighted logistic regression models

Variable	Unadjusted model		Partially adjusted model		Fully adjusted model	
	OR (95%CI)	*P*	OR (95%CI)	*P*	OR (95%CI)	*P*
STB	0.96(0.94,0.97)	< 0.0001	0.96(0.94,0.97)	< 0.0001	0.96(0.95,0.98)	< 0.0001
Stratified by STB tertiles						
Tertile 1	1(Ref)		1(Ref)		1(Ref)	
Tertile 2	0.70(0.60,0.82)	< 0.0001	0.69(0.59,0.82)	< 0.0001	0.73(0.62,0.85)	< 0.001
Tertile 3	0.64(0.53,0.77)	< 0.0001	0.64(0.53,0.77)	< 0.0001	0.69(0.57,0.84)	< 0.001
*P* for trend		< 0.0001		< 0.0001		< 0.0001

*Footnotes* Tertile 1: < 10.26µmol/L; Tertile 2: 10.26–13.68µmol/L; Tertile 3: 13.68–34.2µmol/L. The partially adjusted model was controlled for age, marital status, and race. The fully adjusted model was controlled for age, race, marital status, education, body mass index, smoking, alcohol consumption, diabetes, and hypertension. STB, serum total bilirubin

**Fig. 2:**
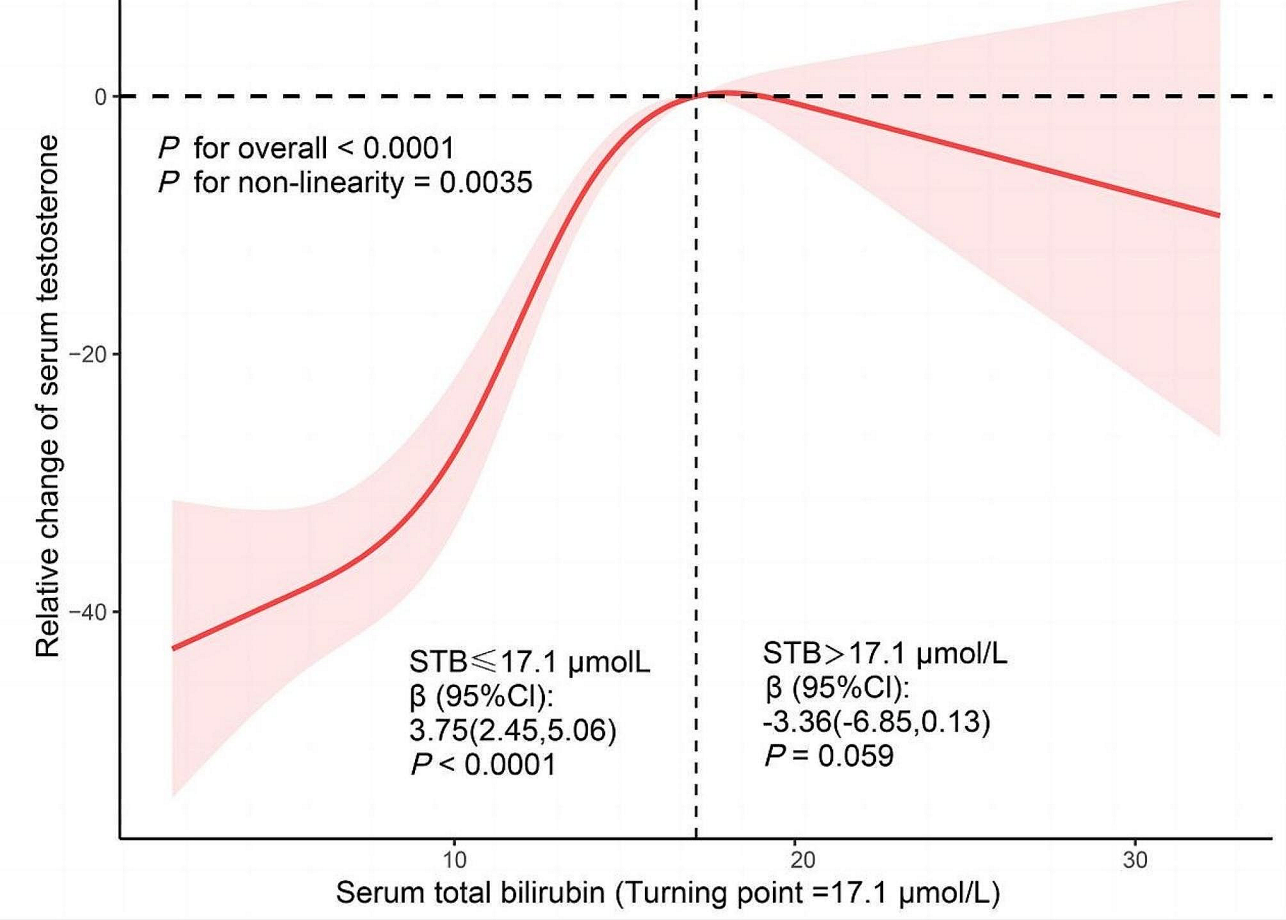
Dose-response relationship analysis between STB and STT. RCS analysis was adjusted for age, race, marital status, education, body mass index, smoking, alcohol consumption, diabetes, and hypertension

The RCS analysis indicated an inverted U-shaped relationship between STB levels and TT, after controlling for age, sex, race, marital status, education level, BMI, smoking status, alcohol consumption, hypertension, and diabetes, with a nonlinear p-value of 0.0035 (Fig. 2). The pivotal threshold was identified at 17.1 µmol/L. For individuals with STB levels ≤ 17.1 µmol/L, a positive correlation with TT was observed, evidenced by a β of 3.75 and a 95% CI of 2.45 to 5.06 (*P* < 0.0001). Conversely, for STB levels > 17.1 µmol/L, the correlation was not statistically significant, indicated by a β of -3.36 (*P* = 0.059). These findings suggest a threshold effect in the relationship between STB and STT levels, underscoring the complexity of their interaction.

### The subgroup analysis and interaction test

**Fig. 3:**
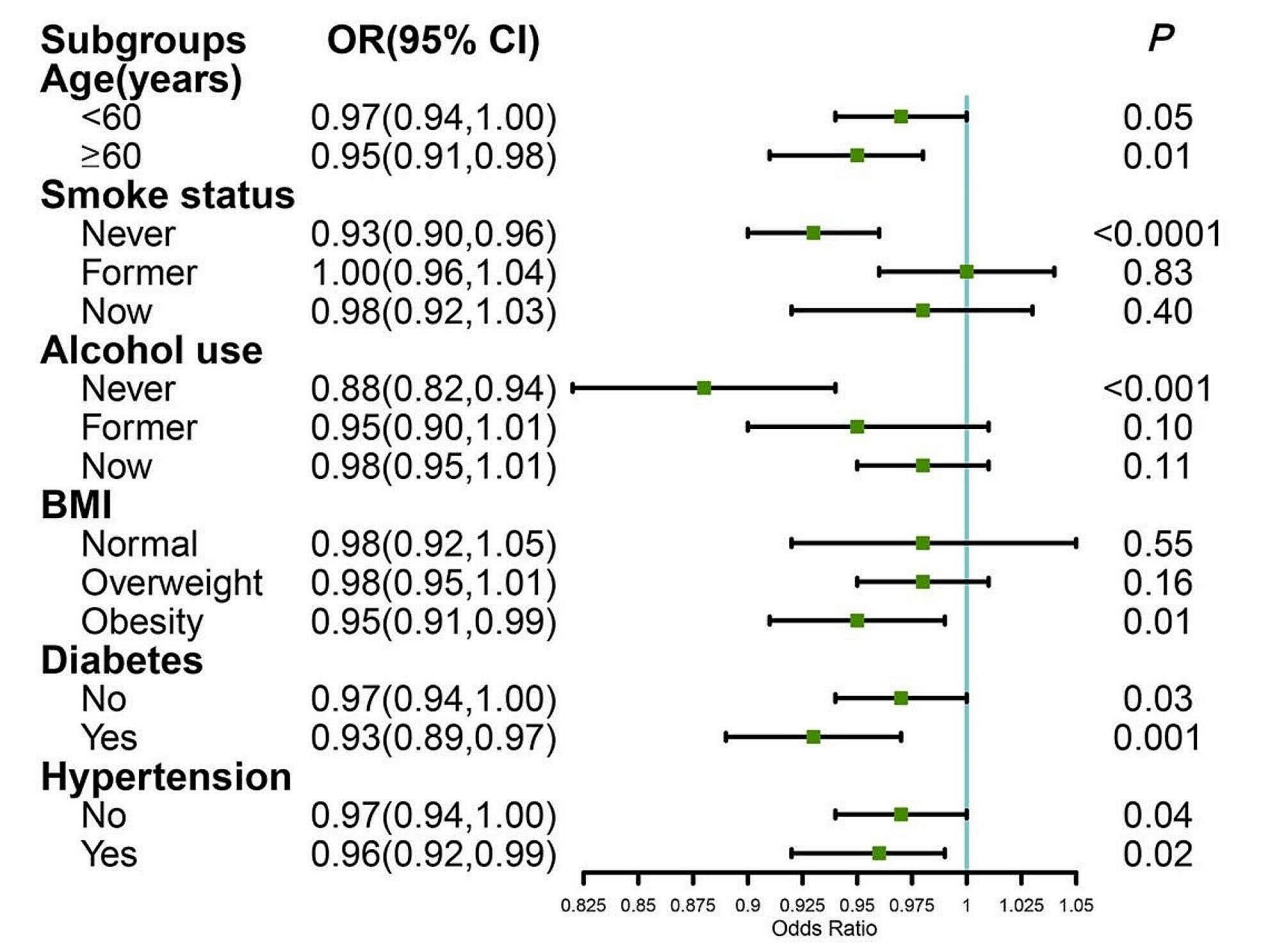
The association between STB (≤ 17.1 µmol/L)and TD in different subgroups. The analysis was adjusted for age, race, marital status, education, body mass index, smoking, alcohol consumption, diabetes, and hypertension. STB, serum total bilirubin

To further explore the relationship between STB (≤ 17.1 µmol/L)and TD occurrence across diverse populations, we conducted a subgroup analysis (Fig. 3). Heterogeneity emerged when stratifying the population by age, smoking status, alcohol consumption, and BMI. Notably, a significant inverse association between STB and TD was observed in individuals over 60 years old, non-smokers, non-drinkers, and those classified as obese (*p* = 0.01, *p* < 0.0001, *p* < 0.001, and *p* = 0.01 respectively). However, among individuals under 60 years of age, former and current smokers, those who had previously or currently consumed alcohol, and those categorized as having a normal BMI or being overweight, no significant association between STB and TD was detected. The respective p-values were 0.05, 0.83, 0.40, 0.10, 0.11, 0.55, and 0.16.

## Discussion

Bilirubin, originating from heme metabolism, is a tetrapyrrole derivative with a highly conjugated structure. Its unique molecular features give it antioxidant properties, enabling it to scavenge free radicals and mitigate oxidative stress. Notably, bilirubin, even when mildly elevated within the normal range, has anti-inflammatory and metabolism-promoting health benefits. Numerous studies confirm that bilirubin significantly reduces the risk of diseases such as obesity, diabetes, and stroke [22, 23]. However, research on bilirubin and TT in serum remains limited. This study explores the preliminary relationship between these two factors. The primary strength of this study lies in its use of national, multiethnic survey data, which facilitates precise analyses stratified by multiple factors and adjusting for various confounders. We found that within the physiological range (0–17.1 µmol/L) [24], STB in adult males shows a positive correlation with TT levels and a negative correlation with the risk of TD (both *P* < 0.0001). STB levels outside the normal range did not exhibit a significant association with TT levels (*P* = 0.059). Previous research on South Korean males also identified a negative relationship between STB levels and the prevalence of TD, although a threshold effect was not observed. Thus, our study provides a more comprehensive analysis of the association between STB and TD.

In our study, the subgroup analysis found an independent and significant negative correlation between STB and TD in individuals over 60 years old. Age-related senescence is the primary risk factor for TD. The decline in testosterone levels with age is linked to Leydig cell aging and impaired testosterone synthesis in older individuals. Older men have fewer Leydig cells compared to younger men [25]. Leydig cells in elderly males show reduced responsiveness to gonadotropin stimulation [26]. The expression of mitochondrial translocator protein decreases in aging Leydig cells, reducing the rate of cholesterol trafficking into mitochondria for TT synthesis [27].

Besides age, obesity is another significant risk factor for a decline in TT levels. Our research indicates a higher incidence of TD in obese men (43.27%) compared to the general population (28.07%). A prospective cohort study involving 3,200 men observed a marked decrease in TT levels among obese individuals [28]. This decline is partially attributed to chronic low-grade inflammation and oxidative stress in obese individuals, which significantly impacts TT levels [29, 30]. Experimental findings have demonstrated elevated levels of oxidative stress markers such as malondialdehyde (MDA) and proinflammatory factors (IL-6 and IL-1β) in the interstitial cells of obese elderly mice [31]. Conversely, antioxidant substances Sirt1 and Nrf2 decrease in senescent interstitial cells [32]. The activation of the p38 MAPK signaling pathway is involved in age- and obesity-induced interstitial cell senescence [31].

Due to its antioxidant and anti-inflammatory properties, mildly elevated bilirubin is beneficial to the human body. Individuals with Gilbert’s Syndrome (GS), characterized by mild bilirubin elevation due to UGT1A1 gene abnormalities, exhibit lower body weight and reduced rates of diabetes and cardiovascular diseases [33, 34]. The role of bilirubin in reducing inflammation and metabolic complications may account for its anti-aging benefits, especially in overweight individuals [35, 36]. Our subgroup analysis revealed a protective effect of STB in preventing TD among obese males over 60 years old. This finding suggests that bilirubin may play a crucial role in maintaining healthier TT levels by mitigating chronic inflammation and oxidative stress linked to obesity and aging.

Furthermore, our findings revealed a significant negative correlation between STB and TD among non-smokers and non-drinkers. The reported effects of smoking on TT levels are inconsistent and contradictory [37]. Some studies have identified a positive correlation between smoking and TT levels [33], while others have observed a non-significant association between the two [38, 39]. Another study found that the positive correlation between smoking and TT weakened in individuals over 60 years old [40]. Our study found that among current or former smokers, subgroup analysis did not reveal a significant inverse correlation between STB and TD, suggesting that smoking may interfere with bilirubin’s protective influence on TT secretion. Additionally, evidence suggests that chronic excessive alcohol consumption could increase the risk of TD [41]. Alcohol consumption elevates oxidative stress, damaging interstitial cells and Sertoli cells, leading to decreased TT levels [42]. Our research highlights the significant protective effect of STB against TD, particularly in non-drinkers, indicating that alcohol consumption may interfere with the relationship between STB and TT. The precise mechanism underlying this relationship remains to be elucidated.

It is essential to recognize certain limitations of our study. The cross-sectional design of the NHANES survey prevents establishing causality between serum bilirubin and TT levels. The absence of data on direct and indirect bilirubin leaves ambiguity about which type is more significantly associated with TT levels. Additionally, the majority of TT is closely bound to sex hormone-binding globulin (SHBG), with only a small fraction existing in a free form with metabolic effects. The assessment of free TT is considered more precise for identifying male TD. However, this survey lacks data on free TT, necessitating further research to investigate its correlation with bilirubin. Furthermore, there may have been insufficient control over potential confounders such as medication use and dietary habits, which could impact bilirubin and TT levels.

## Conclusion

The findings of this study indicate a positive association between STB levels within the normal range and STT levels in adult males. Bilirubin might play a protective role in maintaining normal TT levels in males. Optimizing bilirubin levels through the consumption of natural pigments with similar effects, engaging in regular physical activity, or other interventions may serve as effective preventive strategies to lower the risk of TD. Nonetheless, it is worth noting that our current understanding represents only a fraction of the broader picture. Further studies are warranted to confirm this observation.

## Data Availability

The dataset originates from the National Health and Nutrition Examination Survey (NHANES) and is publicly available on the Centers for Disease Control and Prevention (CDC) website online (https://www.cdc.gov/nchs/nhanes/).
